# Potential long-term impacts of surgical placement cancellations

**DOI:** 10.1080/10872981.2020.1787309

**Published:** 2020-06-28

**Authors:** Chloe Chia, Qi Zhuang Siah, Michael Stephens

**Affiliations:** a School of Medicine, Cardiff University, Cardiff, UK; b Cardiff Transplant Unit, University Hospital of Wales, Cardiff, UK

**Keywords:** Clinical education, career, new technology, professional development, COVID-19

Following the advice [1] released by the Medical School Council in March 2020 in response to the COVID-19 pandemic, medical schools in the UK suspended all remaining clinical placements. Reasonably, these measures aimed to reduce transmission of the virus and ensure student safety. Attempts to replicate surgical placements with e-learning alternatives have not fully replicated the clinical experience, as the practical hands-on approach to learning is irreplaceable (considering the highly physical aspect of surgery). We aim to offer our perspectives as medical students and explore the long-term implications of the cancellation of surgical placements and propose some potential solutions to the issues highlighted.

A surgical placement block typically includes a selection of surgical specialties. Exposure to different surgical specialties varies depending on the specific hospital the student is assigned to, as the availability of different surgical services varies across health boards. There are further variations in surgical placements across different medical schools. Nevertheless, surgical placements provide students with opportunities to develop skills in performing patient examinations and other essential practical skills, as well as develop clinical acumen, all of which are crucial in a junior doctor’s skillset. These opportunities have now been reduced, and students will find an increased difficulty in developing the necessary skills needed to be an effective junior doctor.

A multinational systematic review by Dominic C et al. noted that undertaking a surgical clerkship alone can generate an increased interest in surgery. The opportunity for active participation was also a significant factor in developing an interest in pursuing a surgical career [2]. Berman L et al. found that students who sutured and drove the laparoscopic camera in the theatre were 4.8 times and 7.2 times more likely to be interested in surgery, respectively [3]. Therefore, an absolute lack of surgical exposure could lead to a delayed/missed realisation of one’s interest in surgery, thereby resulting in a potential loss of recruitment of surgeons who have the potential to make significant contributions in the field. It will also result in less time to build evidence of commitment to the specialty.

Loss of recruitment might also have implications on the diversity of the surgical workforce. Diversity in medicine and surgery is important as it leads to more inclusive and patient-centred care, increased patient compliance, satisfaction and participation in clinical trials, and greater access to care for disenfranchised communities [4]. There remains a lack of representation of minority groups in positions of leadership in surgery [5], and mentorship is a crucial factor in promoting a career in surgery [6–10]. Roberts SE et al. found that the perceived barriers preventing African Americans from pursuing a career in surgery included ‘lack of mentorship’, ‘difficulty finding minority role models’, and ‘fighting stereotypes’ [11]. Participants in their study noted the importance of mentors, saying that they felt like they would need mentors to ‘vouch for [them] and to guide [them]’ on the next steps of their careers [11].

Steinberg JR et al. added that underrepresented students are more likely to consider careers that might have previously seemed unattainable or unappealing when they are connected with role models from a similar background [4]. In particular to Oral and Maxillofacial Surgery, Kolokythas A et al. found that increasing the number of female academic surgeons (98%) and having more women in leadership positions (95%) are the most important steps in increasing female involvement in the field [10]. Early exposure to surgical experience in medical school is critical to allow students to build relationships with potential mentors. The cancellation of surgical placements resulted in the loss of the opportunity to network and seek mentorship, which is particularly problematic for those with an intention to pursue a surgical career as it is highly competitive. As undertaking a placement is not feasible in the current climate, these students lost out on opportunities to work and build professional relationships with potential mentors. Recent literature shows that 50% of students sought career advice from mentors who later helped them in deciding their career options [12]. Mentors help students understand their potential, dispel stereotypes, and offer opportunities to develop their interest, knowledge, and experience in the field by allowing students to spend additional time in the surgical department, or to get involved in research. Therefore, a lack of surgical placements could generally hamper students’ future surgical career prospects.

Lastly, there are a few educational aspects of surgical placements which can only be fulfilled in the operating theatre. Students have the unique opportunity to appreciate human anatomy as it relates to surgical pathology and observe different surgical techniques in action. Scrubbing in during procedures comes with additional unique learning opportunities, such as practising the technique for scrubbing in, suturing, and to learn clinical anatomy. This is particularly relevant for medical students who have not had the opportunity to learn via cadaveric dissection, as it provides students with a better frame of reference to place individual organs in relation to each other in space. Online resources are undeniably still helpful, but they can never replicate operating-room based teaching.

Despite the many disadvantages the pandemic has brought, the sudden necessity for remote learning drove the birth of various non-profit initiatives to provide remote teaching for students. Given the capability of engaging in a wider national/international audience via online teaching, there is now the capability of choosing from a more diverse pool of tutors. Furthermore, there remains a role for augmented and virtual reality in surgical education [13], where ideally students would have the opportunity to observe surgical procedures with real-time commentary. Although revolutionary, they are presented with multiple logistical challenges and are yet to be utilised to be part of the curriculum. However, this pandemic might be the catalyst for its implementation in the undergraduate curriculum.

We understand that many of the conventional methods for mentorship and networking are now not feasible. We highlight some potential solutions to the problems we highlighted above:

1. Online conferences to be made free to access, or at a significantly reduced cost of entry, to students
2. Ensure that conference faculty is from a diverse background
3. Host virtual careers evenings or mentorship programs, ensuring that mentors of a diverse background are recruited
4. Host online ‘follow-along’ surgical skills workshops with instructions on how to make homemade low-cost simulation models [14], and allow opportunities for tutors to provide live feedback

Student confidence in performing crucial practical skills needs to be addressed urgently prior to graduation. Schools that organise their surgical blocks for students in a single semester are affected the most, as it will be the only opportunity for students to have dedicated time in surgery. We also highlight the need for additional mentorship opportunities, especially for first-generation and underrepresented minority medical students. In summary, the cancellation of surgical placement blocks, although a necessary step to take to combat the spread of the virus, may have several lasting consequences for medical students.
